# Machine learning-based approach for automated classification of cell and extracellular matrix using nanomechanical properties

**DOI:** 10.1016/j.mtbio.2024.100970

**Published:** 2024-01-20

**Authors:** Tanmay Kulkarni, Olivia-Marie Robinson, Ayan Dutta, Debabrata Mukhopadhyay, Santanu Bhattacharya

**Affiliations:** a Department of Biochemistry and Molecular Biology, Mayo Clinic College of Medicine and Science, 4500 San Pablo Road South, Jacksonville, FL, 32224, USA; b School of Computing, University of North Florida, Jacksonville, FL, 32224 USA; c Department of Physiology and Biomedical Engineering, Mayo Clinic College of Medicine and Science, 4500 San Pablo Road South, Jacksonville, FL, 32224, USA

**Keywords:** Fibrosis, Pancreatic cancer, Extracellular matrix, Nanomechanical attributes, Machine learning, Support vector machines

## Abstract

Fibrosis characterized by excess accumulation of extracellular matrix (ECM) due to complex cell-ECM interactions plays a pivotal role in pathogenesis. Herein, we employ the pancreatic ductal adenocarcinoma (PDAC) model to investigate dynamic alterations in nanomechanical attributes arising from the cell-ECM interactions to study the fibrosis paradigm. Several segregated studies performed on cellular and ECM components fail to recapitulate their complex collaboration. We utilized collagen and fibronectin, the two most abundant PDAC ECM components, and studied their nanomechanical attributes. We demonstrate alteration in morphology and nanomechanical attributes of collagen with varying thicknesses of collagen gel. Furthermore, by mixing collagen and fibronectin in various stoichiometry, their nanomechanical attributes were observed to vary. To demonstrate the dynamicity and complexity of cell-ECM, we utilized Panc-1 and AsPC-1 cells with or without collagen. We observed that Panc-1 and AsPC-1 cells interact differently with collagen and vice versa, evident from their alteration in nanomechanical properties. Further, using nanomechanics data, we demonstrate that ML-based techniques were able to classify between ECM as well as cell, and cell subtypes in the presence/absence of collagen with higher accuracy. This work demonstrates a promising avenue to explore other ECM components facilitating deeper insights into tumor microenvironment and fibrosis paradigm.

## Highlights

• Nanomechanical attributes demonstrate the promise to quantitatively assess fibrosis paradigm using atomic force microscopy.
• Collagen gel’s nanomechanical attributes such as stiffness, deformation and adhesion alter with gel thickness.
• Collagen and fibronectin when mixed in various stoichiometry demonstrates altered nanomechanical attributes.
• Dynamic interactions between pancreatic cancer cells and collagen influence each other’s nanomechanical characteristics.
• Machine learning based techniques successfully classify ECM proteins and pancreatic cancer cells.

## Graphical abstract

**Figure:**
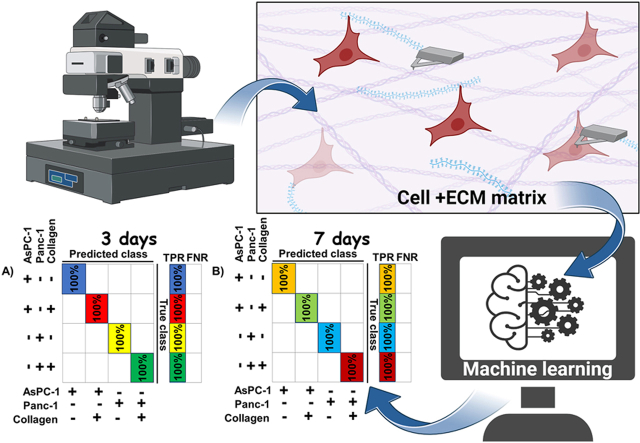

## 1 Introduction

Fibrosis is a hallmark characteristic of various diseases such as liver cirrhosis, pulmonary fibrosis, and myocardial infarction as well as several cancers including pancreatic cancer [[1], [2], [3], [4]]. Fibrotic disorders of the heart, blood vessels, lungs, kidneys, liver, and other organs account for more than a third of annual fatality rates [5]. Fibrosis can be defined as an excess accumulation of extracellular matrix (ECM) components in various paradigms such as healing after injury or pathological processes. Fibrosis progression involves both cell-intrinsic and ECM-driven mechanisms [6]. Cellular intrinsic alterations can initiate a fibrotic response by regulating the differentiation, recruitment, proliferation, and activation of ECM-producing myofibroblasts [7]. Irrespective of triggering events, activation of ECM-producing myofibroblasts is a key mediator of fibrotic tissue remodeling causing fibrotic diseases [5,8]. One of the fallacies restricting the advancement in the fibrosis field is the lack of conceptual knowledge separating fibrosis initiation from fibrosis progression [6]. Bridging this knowledge gap will aid in exploring new therapeutic avenues targeting ECM, disease interventions, and diagnostic regimes.

To study fibrosis, we utilize pancreatic ductal adenocarcinoma (PDAC) as a working model, an aggressive cancer type characterized by severe desmoplasia [9] and immunosuppressive tumor microenvironment (TME) [9,10]. This leads to early-stage metastasis and resistance to chemotherapy [[11], [12], [13], [14], [15], [16], [17], [18], [19]]. Superabundant desmoplasia results from the complex interaction between the ECM and PDAC cells, cancer-associated fibroblasts (CAFs) [10]; and stellate cells [20,21]. Prior research catered to specifically targeting and depleting stromal cells, which only led to further aggravation of the disease [4,[22], [23], [24]]. Following this, the focus shifted to targeting and altering the ECM. Studies have shown that the ECM proteins in TME (tumor microenvironment) can lead to heightened intra-tumoral pressure and prohibit the drug from being delivered to the tumor site [[25], [26], [27], [28]]. Similarly, clinical trials aimed at understanding PDAC ECM have been conducted to no avail [[29], [30], [31]]. Plenty of studies have been undertaken yet, there is a tremendous lack of knowledge associated with fibrotic tumor microenvironment (TME) in PDAC. In PDAC, there is an upregulation in type I, III, and IV collagens as well as fibronectin due to the simulation of stromal fibroblasts by pancreatic cancer cells leading to desmoplasia [32]. ECM has unique abilities to dynamically alter its organization, mechanical properties, and anisotropy. Cells and ECM interact by exchanging mechanical and biochemical forces to generate a conducive environment for disease progression. Thus, it is pivotal to understand the dynamic alteration in nanomechanical properties (NMPs) arising from the complex cell-ECM interactions to gain an insight into fibrosis and its role in disease progression.

Our group has identified major constituents of PDAC ECM components in humans and mice alike [33] such as collagen, fibronectin, and laminin, along with a few more, which in the presence of various cells play a key role in the accumulation of high desmoplasia. Previously, some of the above components have been studied individually, which fails to provide a gross impact in TME [[34], [35], [36], [37]]. In this paradigm, the acquisition of NMPs of various ECM compositions obtained by mixing individual ECM proteins in various stoichiometry±cells will boost our understanding of fibrosis and its role in disease progression. A significant challenge in undertaking this approach to understand cell-ECM interaction is the generation of significant large datasets, which after a point will become hard to manage. Hence, we propose the use of automated machine learning (ML)-based algorithms that can provide an unbiased verdict as opposed to an unintentional bias output potentially resulting from human intervention. We hypothesize that reverse engineering cell-ECM reactions causing dynamic alteration in their NMPs are influenced by the stoichiometry of ECM constituents and cellular varieties. These informative signatures will be detrimental to developing a unique machine learning algorithm to classify fibrotic and cellular regions and identify the state of the pathology.

To realize this hypothesis, we utilized an atomic force microscope (AFM) to evaluate NMPs such as stiffness, deformation, and adhesion of pure as well as mixed ECM components followed by the presence or absence of PDAC cells such as Panc-1 and AsPC-1. The NMP (nanomechanical properties) data was then utilized by an ML (Machine Learning) algorithm to demonstrate various classifications and their corresponding accuracies to exhibit the importance of distinguishing cell-ECM NMPs and ML-driven data classification approaches in this paradigm.

## 2 Materials and methods

### 2.1 ECM gel preparation

To prepare collagen gel samples, Type I Collagen (PureCol® Solution, 3 mg/mL (bovine) #5005) was purchased from Advanced BioMatrix. The 3-D gel was prepared according to the manufacturer's protocol. Briefly, 1 part of chilled 10X phosphate buffer saline (PBS) was mixed with 8 parts of chilled collagen solution with gentle mixing. The resulting mixture is acidic with a pH around 3. To bring the overall pH of the mixture to physiological condition (pH 7.4), we added 0.1 M sterile sodium hydroxide (NaOH). The final volume of the mixture was adjusted to 10 parts using deionized water and the final mixture was stored at 4 °C to avoid gelation. To coat the 60-mm dish, we further diluted the final mixture to prepare 100 μg/mL concentration and incubated at 37 °C for 45 min to form collagen gel. Care was taken such that the collagen solution inside the 60 mm dish was never allowed to dry. To prepare multiple (‘n’ layers) gel layers, the above process was repeated ‘n’ times. After each layer was polymerized, excess collagen solution was aspirated, and fresh collagen solution was added to the plate. Following the desired number of layers, the sample was further processed for characterization or cell culture.

To prepare ECM consisting of collagen and fibronectin, fibronectin (Fibronectin, Solution, 0.5 mg/mL (human) #5050) was also purchased from Advanced BioMatrix and further diluted with cold PBS to reach the same concentration of 100 μg/mL as that of collagen. Following this, they were mixed in various desired parts by volume such as 1:25, 1:50, and 1:75 (fibronectin: collagen), and coated on a 60 mm dish allowing it to incubate at 37 °C for 45 min to form corresponding gel mixtures as mentioned above.

### 2.2 Cell culture

Human pancreatic ductal adenocarcinoma cell lines namely Panc-1 and AsPC-1 were purchased from the American Type Culture Collection (ATCC) and used without further validations. These cells were cultured at 50–60 % confluency on the ECM coated plates in Gibco Dulbecco's Modified Eagle media (DMEM) and Roswell Park Memorial Institute 1640 (RPMI 1640), respectively, and supplemented with 10 % Fetal Bovine Serum (FBS) and 1 % Penicillin Streptomycin at 37 °C in a humidified 5 % CO_2_ atmosphere. After seeding, cells were cultured for 3 and 7 days to facilitate their interaction with the ECM.

### 2.3 Atomic force microscopy

To acquire morphological and nanomechanical traits of ECM and PDAC cells, we employed Dimension Fast Scan with Scanasyst purchased from Bruker Corp. Foremost, the instrument was subjected to optical alignments to maximize signal-to-noise (S/N) ratio. Further, AFM tips were calibrated in a fluid medium to overcome hydrodynamic drag as mentioned in depth previously [38,39]. We employed a pyramidal tip geometry-based scanAsyst Air probe with a nominal spring constant (k) of 0.4 N/m and tip radius of 2 nm for topographical features acquisition. However, for nanomechanical attributes, we incorporated a probe with a 5 μm tip radius and k of 0.1 N/m. Post calibration k was observed to be 0.39 N/m and 0.06 N/m for ScanAsyst Air and 5 μm probe, respectively. In addition, the deflection sensitivity of 130 nm/V was determined for a 5 μm probe. Finally, the sync distance was optimized to occur within ±5 % of the deflection sensitivity at 1 kHz frequency of operation. For the topographical study, a sample size of at least 14 regions was incorporated over three distinct occasions. On the other hand, a sample size of at least 50 data points was considered for the nanomechanical attributes of ECM. For cellular studies, at least 7 cells were selected and probed on 9 distinct regions over the nuclear membrane to accumulate a sample size of 63 data points for each dataset. Force-separation (F–S) curves arising from tip-sample interaction were analyzed using the Derjagin-Muller-Toropov (DMT) model as mentioned previously [38]. Ramping parameters such as ramp size (5 μm), ramp rate (1 Hz), 256 samples/ramp, and an applied trigger of 5 nN for a 5 μm probe) were optimized before data acquisition to generate meaningful data from a completely retracted F–S curve. All the experiments were performed at a physiological temperature of 37 °C, which was maintained using a temperature-controlled AFM stage.

### 2.4 Support Vector Machine (SVM) algorithm

SVM is a non-parametric classification technique, and it relies on a kernel function [40,41]. It can be applied to linearly separable data or to non-linear data. SVM in its original form, like we have used for our experiments, is used as a binary classifier – given an unseen input test data, it predicts whether the data belongs to class A or B. The classification result is deterministic and not stochastic, i.e., it outputs the resultant class with 100 % probability. Unlike standard classification models that try to minimize the error between the observed and the predicted classes, SVM regression aims to fit the best possible line/hyperplane within a known threshold value. However, due to high time complexity, it often gets difficult to train an SVM classification model with a large training dataset. Formally, the training complexity with SVM is O(nd^2^), where n is the number of data points available for training and d is the dimension of the training data. For instance, with three NMPs (stiffness, deformation, and adhesion), d = 3. On the other hand, due to its robustness to outliers (which we have present in our dataset) and high prediction accuracy, we have made it our choice for the classification model.

### 2.5 Data analysis

To analyze the F–S curve, we employed Bruker's Nanoscope v1.9 software. Each F–S curve was foremost baseline corrected followed by curve-fitted using the DMT model to extract nanomechanical parameters. Origin Pro Lab software was used to plot data and perform statistical analysis using the One-Way ANOVA test after confirming the normality criteria were satisfied.

To analyze the performance of the proposed SVM-based classification model, we have used the k-fold cross-validation method. When the available training data size is moderate, k-fold cross-validation is one of the best approaches to check the quality of the developed model. In this, the dataset is first divided into k unique subsets. (k-1) subsets are used in training the model and one subset is used for testing. The process is repeated k times with one of the unique subsets being selected as the test data in every iteration. The average performance measures after the k iterations are reported. The coding of this was done in MATLAB and the results are reported next. In our experiments, k was set to 5.

## 3 Results

### 3.1 The morphology and nanomechanical attributes of collagen gel vary with its thickness

**Fig. 1:**
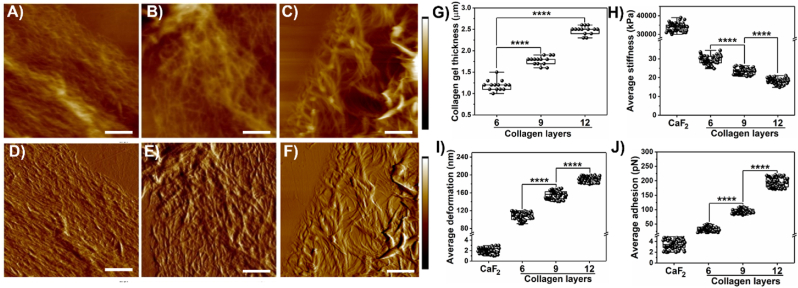
**Nanomechanical attributes of collagen gel layers. Representative morphology images of 6 layers:** A) Height image and B) Peak Force Error (PFE) image, 9 layers: C) Height image and D) PFE image, and 12 layers: E) Height image and F) PFE image. Quantification of collagen gel attributes G) Thickness. H) Stiffness. I) Deformation. J) Adhesion. (Scale bar: 1 μm); (Color bar for A, C and E: 2.5 μm–2.5 μm. Color bar for B, D and F: 1.5 nN–1.5 nN). (n = 14; 1G) and (n = 50; 1H-1J). Statistical significance. ****; p < 0.0001 performed by One Way ANNOVA.

Collagen gel formation exhibits mesh-like topography with intertwined fibers. In a fibrotic paradigm, the thickness of collagen varies based on the degree of pathogenesis [[42], [43], [44]]. Recognizing this phenomenon, we prepared collagen gel samples by polymerizing multiple layers such as 6, 9, and 12 as shown in Fig. 1. The thickness of collagen layers was estimated from the height images (Fig. 1A–C) acquired from the AFM and confirmed to increase systematically with collagen layers as shown in Fig. 1G. Gel thickness for 6, 9, and 12 layers was observed to be 1.19 ± 0.12 μm, 1.77 ± 0.09 μm, and 2.47 ± 0.09 μm, respectively. We observed sufficient mesh structure composed of collagen fibers beginning at layer 6 and progressively thickening with layers 9 and 12 as seen from the peak force error images depicting qualitative information in Fig. 1D–F. Individual fiber thickness was also observed to increase with increasing layers of collagen. We further quantified the nanomechanical properties of collagen gel with varying thicknesses. A polystyrene (PS) dish void of collagen was used as a control and exhibited several thousand-fold elevated stiffness. However, the collagen layer formation expressed a decrease in gel stiffness with the increase in layers as seen in Fig. 1H. Gel stiffness for 6 layers of collagen was observed to be 29.38 ± 2.25 kPa, whereas, for 9 and 12 layers, it was observed to be 23.32 ± 1.53 kPa and 18.23 ± 1.42 kPa, respectively. Deformation exhibited complementary behavior to the gel stiffness as shown in Fig. 1I. As the collagen gel's thickness increased, we observed that the deformation also increased systematically. For 6 and 9 layers, we observed the deformation value to be at 106.64 ± 8.86 nm and 155.22 ± 8.82 nm, respectively. Meanwhile, for 12 layers, deformation was observed to be the most at 191.07 ± 6.22 nm as seen in Fig. 1I. As a control, the PS dish exhibited minimal deformation of 1.98 ± 0.52 nm. Lastly, we also determined the adhesion, which is the repulsive force experienced by the AFM tip as it retracts from the collagen sample surface. It is one of the characteristic attributes of the sample along with stiffness and deformation. This adhesion attribute is different from the adhesion between cell/collagen and the substrate (PS dish).

We observed a methodical rise in adhesion values with an increase in layers. Six layers of collagen gel demonstrated an adhesion value at 33.4 ± 8.86 pN whereas, the control expressed minute adhesion of 3.42 ± 0.85 pN mostly arising from tip and media interactions. With an increase in layers such as 9 and 12, we observed the adhesion value to be at 94.65 ± 7.91 pN and 193.77 ± 16.18 pN, respectively as seen in Fig. 1J. Furthermore, for subsequent characterization of fibronectin-collagen ratios and in the presence of cells, we adapted to 9 layers of collagen gel as a tradeoff between sample preparation time and sufficient gel-like thickness. These results confirm that with an elevated degree of fibrosis characterized by collagen thickness among several other ECM components, nanomechanical and morphological traits are observed to vary and can serve as unique signatures.

### 3.2 Morphology and nanomechanical attributes of ECM mixture vary with stoichiometry change

**Fig. 2:**
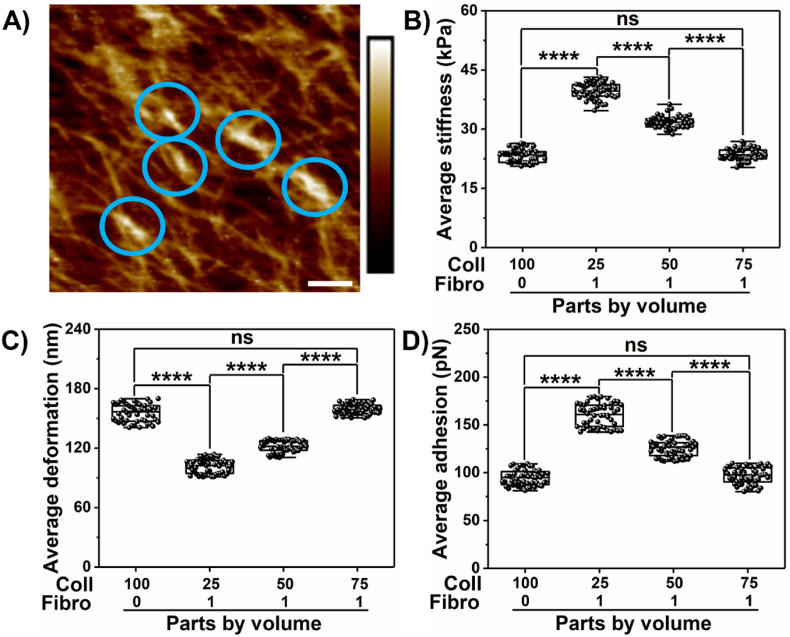
**Nanomechanical signatures of ECM complex.** A) Representative height image of fibronectin and collagen (1:25) (Blue circles indicate fibronectin aggregates). Alteration in nanomechanical attributes for fibronectin and collagen mixed in various stoichiometry ratio, B) Stiffness. C) Deformation. D) Adhesion. (Scale bar: 1 μm). (Color bar −2.5 μm–2.5 μm. (n = 50). Statistical significance: ****; p < 0.0001 performed by One Way ANNOVA.

Apart from type I collagen, fibronectin is also observed to coexist with collagen and has implications in desmoplasia. Fibronectin is observed to form aggregates instead of a mesh structure [45,46]. However, fibronectin globules bind to collagen fibers as seen from the height profile image in Fig. 2A. These are shown by the encircled regions indicated by bright colors exhibiting elevation. We further explored the influence of fibronectin on nanomechanical attributes of fibronectin-collagen mixtures when amalgamated in various stoichiometry ratios as shown in Fig. 2B–D. We observed that by mixing 1 part of fibronectin with 25 parts of collagen, the stiffness of the mixture was measured to be 39.63 ± 2.06 kPa, which was significantly more than 9 layers of collagen alone as seen in Fig. 2B. With increasing collagen content in the collagen-fibronectin mixture, we observed that the overall gel stiffness decreased until it was insignificant compared to 9 layers of collagen alone. Mixture stiffness was observed to be 31.93 ± 1.55 kPa and 23.51 ± 1.5 kPa for fibronectin to collagen when mixed at 1:50 and 1:75, respectively. The mixture of fibronectin to collagen at 1:75 exhibited an insignificant alteration in stiffness compared to 9 layers of collagen alone. It suggested that at this ratio, the contribution of fibronectin within the mixture was minimal and the overall mixture behaved similarly to stand-alone collagen as seen in Fig. 2B. The deformation trend was observed to be opposite to the mixture stiffness. With the highest mixture stiffness observed at fibronectin to collagen at 1:25, deformation was observed to be the least at 101.77 ± 7.18 nm. However, with the mixture ratio at 1:50 and 1:75, deformation was observed to increase to 121.83 ± 5.88 nm and 159.2 ± 5 nm, respectively. The adhesion parameter was also observed to systematically increase with the increase in the collagen content of the mixture and was observed to be 96.74 ± 8.72 pN, 125.44 ± 8.39 pN, and 160.34 ± 11.86 pN for 1:25, 1:50 and 1:75 parts by volume of fibronectin is to collagen, respectively. These results confirm that nanomechanical attributes of ECM alter with variation in the stoichiometry of its constituents.

### 3.3 PDAC cellular presence alters collagen's nanomechanical attributes dynamically

**Fig. 3:**
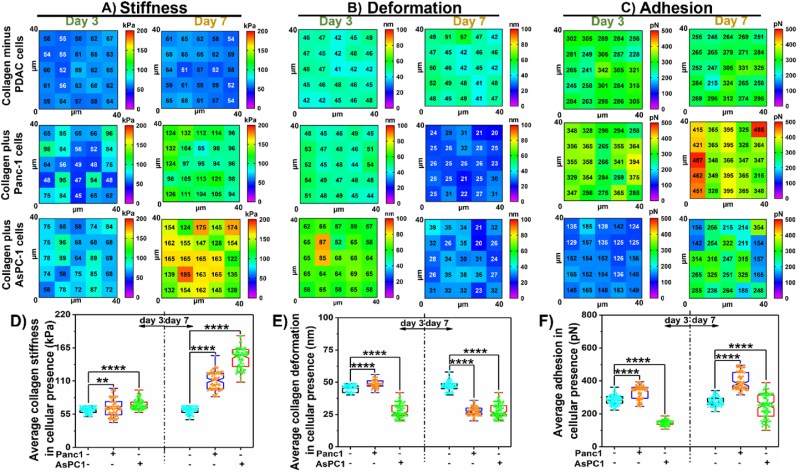
**Representative heat map demonstrating dynamic alteration in nanomechanical attributes of collagen in the presence or absence of PDAC cells over 3 and 7 days.** A) Stiffness. B) Deformation. C) Adhesion. **Quantification of nanomechanical attributes of collagen in the presence or absence of PDAC cells over 3 and 7 days.** D) Stiffness. E) Deformation and F) Adhesion. (n = 125) Statistical significance. ****; p < 0.0001 performed by One Way ANNOVA.

In the PDAC paradigm, severe desmoplasia is the result of interaction between various cells and ECM [21,47]. Both components regulate the conducive environment for the disease to thrive through mutual assistance [48,49]. Hence, it becomes imperative to study the influence of cellular presence on collagen for dynamic time durations. For proof of principle experiment, we evaluated nanomechanical attributes of collagen in the presence and absence of PDAC cells such as Panc-1 and AsPC-1. For this study, we used a bead with a 5 μm tip radius for an easy transition to analyze tissue sections in the future. To avoid redundancy in data acquisition using large beads for various nanomechanical attributes, we determined that 8 μm is a sufficient distance between adjacent data points. Thus, on a 40 × 40 μm^2^ region, we were able to acquire 5 x 5 pixels (datapoints) indicated by the heat map in Fig. 3. In the absence of PDAC cells, collagen's nanomechanical attributes remain unaltered over 3 and 7 days as seen from the top panel in Fig. 3A–C. However, we observed that between 1 and 3 days, its stiffness increased ∼2.5-fold to an average of 61.70 ± 4.61 kPa compared to 23.32 ± 1.53 kPa as seen from Fig. 1, Fig. 3D. Similarly, deformation was observed to reduce ∼3-fold times to 45.21 ± 2.55 nm at the end of 3 days compared to 1 day, which was equivalent to 155.22 ± 8.82 nm as observed in Fig. 3B–E. We also observed a ∼3-fold increase in average adhesion from 94.65 ± 7.91 pN at 1 to 284.72 ± 28.15 pN at 3-day time duration as seen from Fig. 3C and F. The presence of cells further influenced the nanomechanical attributes of collagen dynamically. For instance, in the presence of Panc-1 cells, at day 3, collagen's average stiffness increased slightly to 66.28 ± 14.76 kPa compared to the absence of cells. Deformation and adhesion also increase slightly to 48.68 ± 3 nm and 329.84 ± 41.72 pN, respectively compared to day 1 as seen from the middle panel in Fig. 3A–C and 3D-3F. However, more significant alterations were observed on day 7 compared to day 3. Collagen's average stiffness increased 1.8-fold times to be at 110 ± 14.54 kPa whereas, its deformation decreased significantly by 1.67-fold to be at 27.43 ± 3.55 nm. Additional heat maps of nanomechanical attributes of collagen with and/or without PDAC cells displaying uniformity in the attribute's distribution are shown in Figs. S2–S4. Adhesion attribute increased by 1.45-fold to be at 397.79 ± 53.14 pN, compared to collagen's nanomechanical attributes in the absence of PDAC cells as seen from Fig. 3A–C: middle panel and 3D-3F.

We also monitored the dynamic alteration of collagen's nanomechanical attributes in AsPC-1 cells. At day 3, collagen stiffness and deformation rose to 71.09 ± 7.33 kPa and 59.81 ± 6.13 nm respectively as seen from the bottom panel in Fig. 3A and B, whereas its adhesion decreased significantly to be at and 148.03 ± 14.61 pN compared to collagen's nanomechanical attributes in the absence of PDAC cells as seen from the bottom panel in Fig. 3C and F. At 7-day time duration in the presence of AsCP-1 cells, collagen's stiffness increased by 2.41-fold (Fig. 3A: bottom panel) and 3D, while its deformation and adhesion decreased by 1.7-fold and 1.11-folds (Fig. 3B and C: bottom panel and 3E and 3F), respectively compared to the absence of PDAC cells. Cumulative average values of nanomechanical attributes of collagen with or without PDAC cells are shown in Fig. 3D–F. These results suggest that the genetic signature of induvial cell type certainly has a distinct impact on the alteration of collagen's nanomechanical properties.

### 3.4 Collagen influences dynamic alteration in nanomechanical attributes of PDAC cells

**Fig. 4:**
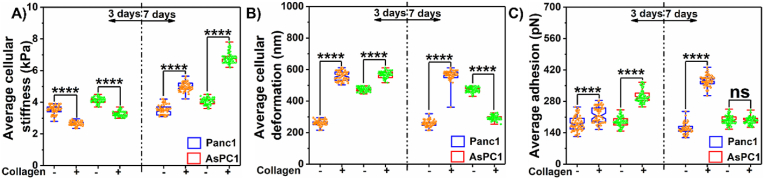
**Dynamic influence of collagen on nanomechanical attributes of PDAC cells.** A) Stiffness. B) Deformation and C) Adhesion. Statistical significance. ****; p < 0.0001 performed by One Way ANNOVA.

Since there is active crosstalk between cells and ECM in the progression of PDAC, we also examined the influence of collagen on nanomechanical attributes of PDAC cells over 3 and 7 days, respectively. As a control, we cultured PDAC cells without collagen for respective durations and monitored their nanomechanical attributes. Panc-1 cells without collagen at 3 days exhibited membrane stiffness of 3.5 ± 0.25 kPa (Fig. 4A) with deformation and adhesion of 261 ± 16.7 nm and 181.65 ± 32.29 pN, respectively (Fig. 4B and C). AsPC-1 cells at the end of day 3 exhibited average membrane stiffness, deformation, and adhesion corresponding to 4.13 ± 0.19 kPa (Figs. 4A), 475.73 ± 14.03 nm, and 188.31 ± 21.56 pN, respectively (Fig. 4B and C). These nanomechanical attributes remained unaltered at day 7 as seen from Fig. 4A–C. However, in the presence of collagen, we observed that the Panc-1 and AsPC-1 cells appeared softer bearing membrane stiffness of 2.63 ± 0.13 kPa and 3.28 ± 0.17 kPa, respectively as seen in Fig. 4A. Deformation on the other hand appeared to increase significantly compared to the absence of collagen on day 3. Panc-1 and AsPC-1 cells exhibited deformation of 559.36 ± 29.66 nm and 569.01 ± 20 nm, respectively as seen in Fig. 4B. Adhesion was also elevated due to collagen in both PDAC cells. We observed the adhesion to be at 221.34 ± 34.93 pN and 303.28 ± 28.97 pN, respectively for Panc-1 and AsPC-1 cells as seen in Fig. 4C.

At a 7-day time duration, we observed an opposite trend in membrane stiffness in both Panc-1 and AsPC-1 cells compared to day 3. We observed an increase in membrane stiffness at 4.96 ± 0.25 kPa and 6.82 ± 0.37 kPa for Panc-1 and AsPC-1 cells, respectively (Fig. 4A). While deformation for Panc-1 showed no change compared to day 3, in the case of AsPC-1 cells, the deformation parameter dropped significantly and was observed to be at 294.29 ± 14.17 nm as seen in Fig. 4B. Adhesion in the case of AsPC-1 cells showed no significant alteration between the presence and absence of collagen on day 7. However, in the case of Panc-1, we observed a significant increase in its value corresponding to 373.8 ± 23.83 as seen in Fig. 4C. This data proves quantitatively for the first time that cells and ECM indeed have influence over each other's nanomechanical attributes and vary dynamically.

### 3.5 Dynamic alteration of morphological characteristics of cells in the presence of collagen and their correlation with nanomechanical attributes

**Fig. 5:**
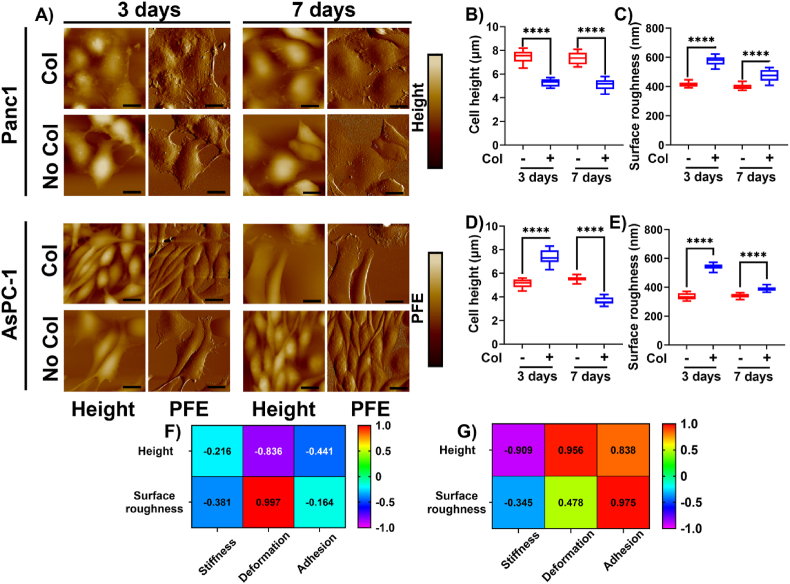
**Dynamic alteration in morphological traits of PDAC cells in the presence and absence of collagen.** A) Representative height profile and peak force error (PFE) images of Panc1 and AsPC-1 cells in the presence and absence of collagen. Quantification of B) Height and C) Surface roughness in Panc1 cells. Quantification of D) Height and E) Surface roughness in AsPC-1 cells. Heat map exhibiting correlation coefficients between morphological and nanomechanical attributes in F) Panc1 and G) AsPC-1 cells. In Figure A: Scale bar: 2 μm; (Color bar -9μm–9 μm for height profile images and from -5nN to 5 nN for PFE images. (n = 12). Statistical significance. ****; p < 0.0001 performed by One Way ANNOVA.

Prior research has demonstrated the relation between actin reorganization and cellular stiffness [50 51]. Herein, we observed a dynamic alteration in the cellular nanomechanical attributes of PDAC cells in the presence and absence of collagen. Instead of pinpointing various cytoskeletal components, we monitored key morphological traits namely, cellular height and surface roughness. Surface roughness has been associated with various cytoskeletal components such as Actin, bilipid layers, etc.[52 53]. Fig. 5A shows representative height profile and peak force-error images of Panc-1 and AsPC-1 cells in the presence and absence of collagen cultured over 3 days and 7 days' time. The height profile yields overall height as well as the surface roughness over the nuclear membrane region of the cells. Peak force error images, however, result from the feedback error the detector receives while the scanning process provides qualitative images. We observed that cellular height in Panc1 cells was unaffected in the absence of collagen as seen from Fig. 5B. However, in the presence of collagen, we observed a decrease in cellular height compared to its control counterparts. Cell height for Panc-1 cells in the absence of collagen over 3 and 7-days' time was 7.5 ± 0.5 μm and 7.4 ± 0.51 μm, respectively. In the presence of collagen, height values were observed to be significantly lower than the control and were 5.3 ± 0.33 μm and 5.13 ± 0.45 μm post 3 and 7 days of culture, respectively. On the other hand, surface roughness was observed to be increased in the presence of collagen and was determined to be 575.58 ± 31.55 nm and 473.16 ± 40.72 nm post 3 and 7 days of culture compared to corresponding control (without collagen) 416.41 ± 16.55 nm and 400 ± 19.8 nm, respectively as seen from Fig. 5C. In the AsPC-1 cell lines, at 3 days of culture, cellular height was observed to increase in the presence of collagen compared to the absence and the corresponding values were observed to be 7.35 ± 0.63 μm and 5.15 ± 0.34 μm, respectively. However, at 7 days of culture, we observed the cellular height in the presence of collagen to be significantly lesser than absence of collagen with values corresponding to 3.64 ± 0.33 μm and 5.25 ± 0.21 μm, respectively as seen from Fig. 5D. The surface roughness followed a similar trend as Panc-1 with values significantly more in the collagen's presence than non-collagen. At 3-day time, we observed that the surface roughness of non-collagen cultured AsPC-1 cells was 337 ± 22.65 nm compared to the collagen cultured cells at 541.58 ± 21.7 nm. Whereas, at day 7 of the culture, we observed the surface roughness of the AsPC-1 cells in the absence of collagen was 340.58 ± 14.83 nm compared to its collagen counterpart with 387.5 ± 15.47 nm as seen from Fig. 5E. Finally, we determined if the morphological traits bore any correlation with the nanomechanical attributes by performing Pearson's correlation. The results were plotted in the form of heat map with correlation coefficient values ranging from +1 to −1 (directly correlated to inversely correlated) with values closer to ‘0’ indicating minimal correlation. In the case of Panc-1 cells in Fig. 5F, we observed that the height and surface roughness were negatively and positively correlated with deformation attribute with coefficient of −0.836 and 0.997, respectively. In AsPC-1 cells, height profile was better correlated with all three nanomechanical attributes such as stiffness, deformation, and adhesion with coefficients of −0.909, 0.956 and 0.838, respectively as seen from Fig. 5G. The surface roughness, however, was only correlated with adhesion bearing the coefficient of 0.975. These correlation coefficients suggest a link between nanomechanical and morphological attributes and needs to be pursued further.

### 3.6 ML-based classifier segregates cell and ECM

We used accuracy as our primary performance metric to quantify the quality of the proposed SVM-based classification models. The accuracy of a classification model is defined as follows.

(1) $$Accuracy=\frac{number\;of\;correct\;classifications}{number\;of\;test\;inputs}*100$$

Thus, the higher the accuracy, the better the model is correctly classifying between two or more classes (e.g., ECM and cell).

Foremost, we were interested in finding out the accuracy of the prediction of whether the input AFM data is coming from a cell or collagen. Our proposed SVM (Support Vector Machine) model with a linear kernel provided 100 % accuracy. In Fig. S1, the confusion matrix – where the number of test instances for the cell and collagen classes is seen to be always correctly classified as cell and collagen, respectively.

**Fig. 6:**
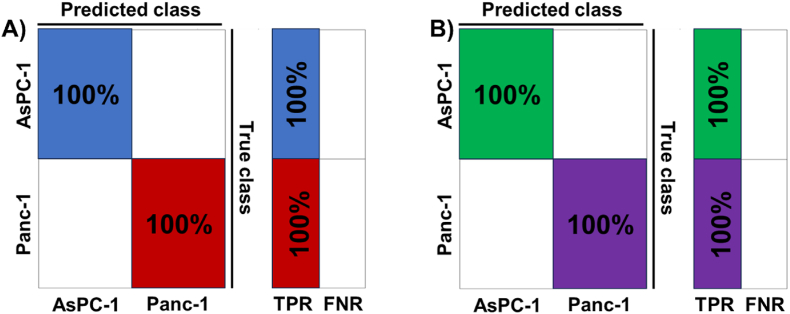
**ML technique classifies Panc-1 and AsPC-1 cells in the absence of collagen based on their nanomechanical attributes.** SVM based classifier with a linear kernel was employed. A) 3 days of culture and B) 7 days of culture.

Following this, we investigated how the interaction between cells and collagen affected the accuracy of the ML-based classification solution. We had already established that PDAC cellular presence altered collagen's AFM attributes. The question now was whether an automated ML-based classifier could identify this phenomenon. As a baseline, we first investigated the scenario where there was only cell present with no collagen. We hypothesized that an ML-based classifier can automatically classify between the two test cell lines, namely Panc-1 and AsPC-1. Based on the AFM data of cells only, we achieved a perfect, 100 % accuracy in differentiating between the two cell lines when there was no collagen present in either the 3- and 7-day scenarios as seen in Fig. 6A and B. In both these results, we used a linear kernel for the SVM-based classifier. The confusion matrices are presented in Fig. 6A and B.

**Fig. 7:**
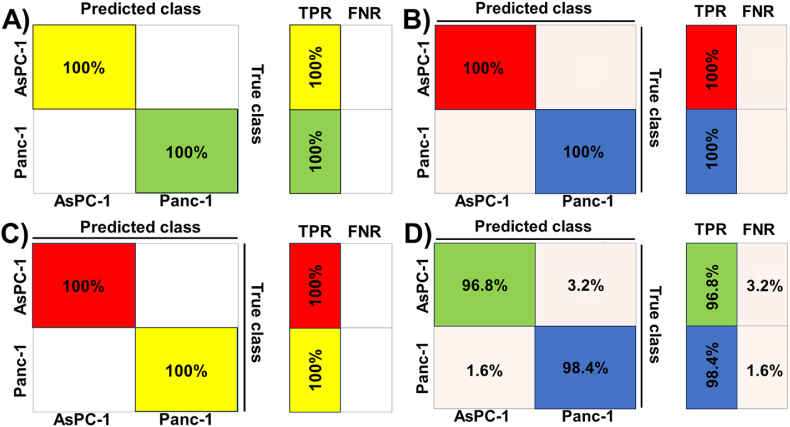
**ML technique classifies Panc-1 and AsPC-1 cells in the presence of collagen based on their nanomechanical attributes.** SVM based classifier with a linear kernel was employed for both, A) 3 days of culture and B) 7 days of culture. **ML technique classifies collagen in the presence of Panc-1 and AsPC-1 cells based on their nanomechanical attributes.** SVM-based classifier with a linear kernel was used for C) 3 days of culture and SVM-based classifier with a quadratic kernel for D 7 days of culture.

Next, we investigated the case when cells were present in collagen. For this, we considered the presence of Panc-1 and AsPC-1 cell lines in collagen for 3 and 7 days. Using a linear kernel-based SVM, we achieved 100 % accuracy in classifying the cell lines (Panc-1 or AsPC-1) for both 3- and 7-day scenarios. Fig. 7A and B presents the corresponding confusion matrix. Further, we focused on whether a traditional ML-based classification model could classify between collagen in the presence of Panc-1 and AsPC-1 cell lines. We observed that for the 3-day case, the SVM model with a linear kernel could yield 100 % accuracy whereas in the 7-day scenario, the accuracy in predicting the cell line correctly was high at 97.6 %. Furthermore, in this case, collagen in the presence of AsPC-1 was predicted as collagen in the presence of Panc-1 3.2 % times whereas the collagen in the presence of Panc-1 cell was incorrectly classified as collagen in the presence of AsPC-1 cell 1.6 % times. Note that we used a quadratic kernel for this case (Fig. 7D). The corresponding confusion matrices are presented in Fig. 7C and D.

**Fig. 8:**
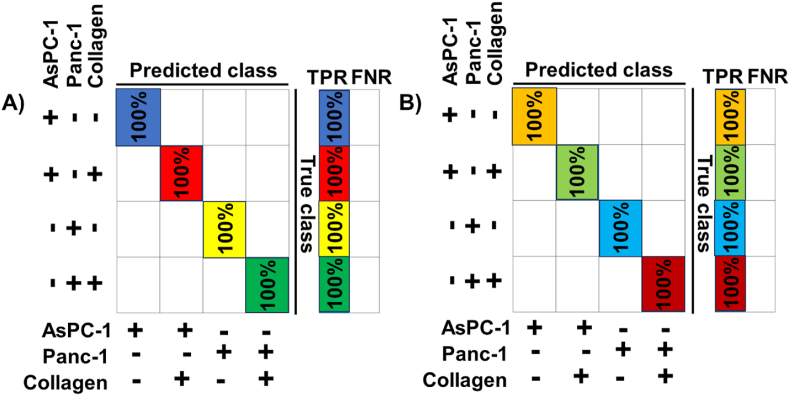
**ML technique classifies Panc-1 and AsPC-1 cells in the presence and absence of collagen based on their nanomechanical attributes.** SVM based classifier with a linear kernel was employed. A) 3 days of culture and B) 7 days of culture.

The next research question we posed was whether such an SVM-based classifier could correctly classify data into four classes – namely, Panc-1/AsPC-1 cells and Panc-1/AsPC-1 cells in collagen. Like the previous experiments, we performed both 3- and 7-day classification studies to answer this question. The results are presented in Fig. 8A and B. We observed that our proposed ML-based classifier always yielded 100 % accuracy in correctly classifying the class of the input sample highlighting the strength of the automated ML-based classifier.

**Fig. 9:**
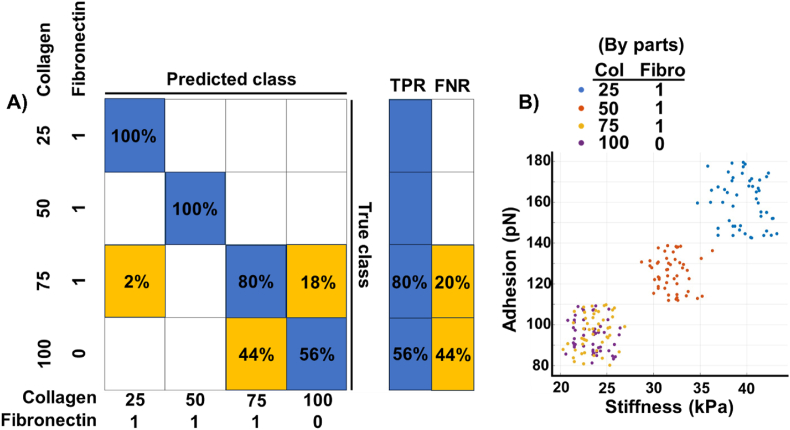
**ML technique classifies collagen and fibronectin stoichiometry ratios based on their nanomechanical attributes.** SVM based classifier with a linear kernel was employed. A) Confusion matrix exhibiting true positive rate (TPR) and false negative rate (FNR). B) Scatter plot depicting adhesion and stiffness values for various stoichiometry ratios of collagen and fibronectin shows a clear overlap between collagen and 1:75 by parts of fibronectin and collagen.

We previously discussed how the morphology and nanomechanical attributes of the ECM mixture vary with stoichiometry change. In terms of experimenting with the classifier model, we were interested in investigating whether this variance in the AFM data can be captured by the SVM classifier. To test this, we set the four output classes to be collagen and three types of fibronectin-collagen mixtures (1:25, 1:50, and 1:75). We used a Gaussian kernel for this usage of SVM, which yielded the best accuracy results. The average accuracy was found to be 84 %. The confusion matrix is presented in Fig. 9. The dip in classification accuracy mostly comes from two classes – collagen and a fibronectin-collagen 1:75 mixture. Collagen was misclassified as the said mixture 44 % time whereas the fibronectin-collagen mixture was misclassified as collagen 18 % time. To dig deeper into this, we investigated the input AFM features for the classes. As seen from Fig. 1, Fig. 2D, either of the AFM properties (stiffness and adhesion) could distinctively classify between collagen and the 1:75 mixture visually while the other two types of mixtures were distinctive enough. This consequently allowed the SVM classifier to incorrectly classify between collagen and the 1:75 mixture of fibronectin-collagen and yielded an average of 32 % false negative rate between them. As the collagen proportion in the stoichiometry ratio increases, the mixture behaves more like collagen alone and hence, the inaccurate classifications.

## 4 Discussion

Fibrosis is a hallmark characteristic in several diseases including pancreatic cancer. In PDAC, it is known as desmoplasia, a dense cluster of various cells including cancer and intense ECM. Owing to high desmoplasia, traditional therapies seldom improve the survival rate in patients. This has prompted researchers to explore novel therapeutic avenues to improve the 5-year survival in patients and their overall standard of living. However, we still lack significant knowledge governing the TME, especially the interactions between cells and ECM leading to a conducive environment for the disease to thrive. In this work, we focused on collagen and fibronectin, which are abundantly present in desmoplastic environments along with PDAC cells such as Panc-1 and AsPC-1 and explored their influence on each other in terms of nanomechanical attributes. We observed that collagen gel thickness drives its nanomechanical attributes and the addition of fibronectin to collagen in various stoichiometry ratios further alters nanomechanics. By increasing the number of collagen layers, we achieved a denser mesh with enhanced thickness as shown in Fig. 1A–G. Previously, it has been shown that a higher density of collagen increased tumor density and altered its mechanical traits compared to normal pancreatic tissue [50]. Our results agree with prior observation and indeed alter mechanical traits. Fibronectin is distributed between collagen fibrils according to a previous study [51]. Fibronectin is known to accelerate the fibrillogenesis of type I collagen and could lead to enhanced ECM stiffness as observed in Fig. 2. Furthermore, the presence of cells enhanced the stiffness of collagen several folds along with alterations in other nanomechanical attributes over 7 days. Conversely, the presence of collagen made the cells softer initially at the end of 3 days but later made them stiffer over 7 days compared to cells without collagen's presence. Moreover, AsPC-1 cells exhibited opposite trends in deformation attributes over 3 and 7-day periods in the presence and absence of collagen. Similarly, the adhesion parameter was also altered for both cells over 7-day periods except for AsPC-1 cells on day 7. These results confirm the dynamic nature of cell-ECM interactions within the PDAC regime and serve as a promising avenue to explore alterations in the presence of other cells and ECM components as well.

The dynamic reciprocity between cell-ECM is enhanced in pathogenesis [52]. In the cancer paradigm, fibrotic and desmoplastic environments motivate myofibroblast activation [53]. These myofibroblasts deposit uncontrollable amounts of ECM leading to severe desmoplasia. In response, cellular activity is affected by altering the cytoskeleton, which drives the secretion of matrix-remodeling molecules such as collagen and MMPs [54]. Remodeling of ECM prompts a broad range of biophysical and biochemical alterations that further induce cascade effects in cell signaling, cell migration, and tumor progression [55]. Several 3D models have been studied previously as they recapitulate key tumor microenvironment signatures [56 57]. For instance, Liu et al. introduced a novel method of preparing collagen I based ECM structure by mechanical agitation method [56]. Such a scaffold appeared thick and wavy in addition to preserving the global softness and resembled collagen architecture in the tumor stroma. It further promoted tumor cell dissemination, triggering differences in morphology and migratory behaviors of tumor cells. In another instance, Nguyen et al. developed a method to initiate formation of collagen islands possessing highly tunable inclusions and mechanical attributes in a collagen hydrogel [57]. It was used to demonstrate alteration in cell migration and osteogenic differentiation of mesenchymal stem cells. Additionally, the collagen island architecture was able to induce mesodermal differentiation in pluripotent stem cells [57]. While such complex and enhanced 3D models are already in practice, we desire to undertake an extremely basic approach that will enable us to gain insights into the cell-ECM dynamics at a grass root level. This will let us develop a robust ML algorithm by blending in various other ECM and cellular components stepwise. Post generation of nanomechanical attributes library with basic ECM building blocks, we plan to perform validations in complex 3D ECM models. We observed that the PDAC cells first appeared softer on day 3, followed by them being stiffer on day 7 compared to the cells without collagen. This could be attributed to the complex and dynamic cell-ECM interactions. Initially, the cells could rest on top of the ECM and be exposed to fluid media. At this early instant, the cells could begin to settle in harmony with the ECM and appear labile, hence the softer cells. However, in 7 days, these cells could be well settled in the presence of the ECM as well and there might be a thin layer of ECM formed on the cells, which is known to increase the cell stiffness [58]. Another plausible explanation is that with prolonged exposure to collagen, the cellular cytoskeleton could have undergone dynamic alteration resulting in enhanced stiffness as we observed in the morphological attributes in Fig. 5A–E. We further quantified morphology attributes such as cell height and nuclear membrane roughness to recapitulate various cytoskeletal elements that may undergo alterations in the presence and absence of collagen dynamically in a temporal manner. Based on the data we further performed Pearson's correlation between these attributes and nanomechanical attributes under same treatment conditions. We observed that there are significant correlations between certain parameters, thus demonstrating promise. Currently, with the simplistic model, the ML algorithm was able to classify various conditions accurately solely based on nanomechanical attributes. However, in the future with the inclusion of more ECM and cellular components, which will elevate the complexity of the model, we could certainly include morphological traits as additional differentiating signatures or even use the correlation criteria as one of the distinguishing parameters to classify such components. Lastly, based on the study performed by Michael Mak in which, the author via computer simulations of 3D ECM fiber networks demonstrated that ECM composition crosslinks are detrimental to the degree of densification and stiffening influenced by the forces exhibited by the cells [59]. In our study, we observed that stiffness profile of the Panc1 cells at day 3 and day 7 were opposite to each other. This could be attributed to the varying ratio of permanent crosslinks to the total crosslinks as studied previously [59]. Higher ratio demonstrated reduced accumulation of ECM toward the cell, which the accumulated ECM was reversed after unloading of the force, exhibiting elastic recovery. At the same time, networks with lower ratio exhibited high accumulation of ECM in a nonelastic manner [59]. We also observed that the stiffness of collagen enhanced multi-fold with an increase in the time duration of cellular exposure. The collagen fibers that maintain structural integrity may hold to different cells or similar interconnected fibers. This results in significant tensile strength along the fibers and hence, enhanced collagen stiffness in the cellular presence. This behavior is also observed in 3D spheroid models studied previously [58]. We also observed that the stiffness of collagen increased at day 7 compared to day 3 in the presence of PDAC cells. Prior studies indicated that ECM network experiences increase in tension, which relaxes over time due to lower ratio of permanent to total crosslinks in ECM matrix, which was opposite to the results obtained here [59].

Several shapes and sizes of the AFM tips are employed to study various biological samples [39,60]. To evaluate the topographical alterations in samples, sharper tip-containing probes are preferred as they assist in acquiring high-resolution topographical images due to their minimal surface area for interaction [61]. On the contrary, nanomechanical attributes are acquired using a spherical tip-containing probe with a diameter of several μm to prevent the tip from penetrating the sample surface and thus preserving its integrity [39,62]. F–S curves from which nanomechanical attributes are extracted by curve fitting with various contact mechanics models and it is imperative that the tip completely retracts from the sample post indentation [39,63]. Moreover, the utilization of a 5 μm bead will assist in an easy transition to tissue samples to study the ECM-cell interactions. Previously, AFM was employed to study the elastic properties of single collagen type I fibrils [64] as well as the topography of collagen gel [65]. Similarly, fibronectin has been studied distinctly in which, the authors observed a high extensibility in fibronectin due to unfolding of its FNIII domains [66]. These domains required 80 pN and 200 pN of force to unfold the weakest and strongest domains, respectively. In the presence of cells, collagen-film interaction has been studied before. For instance, the D-band periodicity has been shown to play a vital role in collagen-cell interactions as cells respond to information contained in this periodic pattern [67]. Morphological studies including cellular presence on collagen coating suggested that cells frequently align themselves with the fibrils indicating fibrils provide crucial signaling cues for cell polarization [68]. Moreover, cell polarization and directional cellular traction were influenced by the high tensile strength and pliability of collagen fibrils [68]. In terms of fibronectin's interaction with cell surface, fibronectin coated AFM bead probe approached mouse fibroblast cell surface demonstrated a higher mean separation work (∼4-fold more) compared to the unmodified tip and can serve as an effective model to study adhesive forces on the cell surface [69]. These segregated studies qualitatively or quantitatively study various parameters involved in cell-ECM interaction but fail to provide the dynamic alterations in nanomechanical attributes resulting from stoichiometry combinations of ECM, which this work strives for.

Prior studies have been undertaken in the application of AI/ML to gain insights into various pathogenesis [[70], [71], [72], [73], [74]]. For instance, amalgamation of Pan-Cancer expression data from The Cancer Genome Atlas (TCGA) with genomics, epigenomics and microenvironmental signatures enabled the authors to identify specific genes involved in genetic and epigenetic programing of the tumor matrisome and led to development of novel therapeutic avenues [70]. Another study focused on the deep learning-based classification of morphometric parameters from multi-channel fluorescence micrographs of fibroblasts and epithelial cells upon exposure to different ECM. Their study demonstrated the non-random nature of cell shape and established the framework for classifying cell shapes into distinct morphological signature in a cell-type and ECM-specific manner [71]. More recently in breast cancer paradigm, self-organizing maps (SOMs) as unsupervised artificial neural network was applied to distinguish between estrogen treated, control and resveratrol treated cells in an unsupervised manner based on cell stiffness and viscosity [72]. To understand the underlying mechanisms behind idiopathic pulmonary fibrosis (IPF), Luo et al. developed an ECM-related risk model utilizing Least Absolute Shrinkage and Selection Operator (LASSO), Random Forest, and Support vector machines algorithms. Based on various genes such as CST6, PPBP, CSPG4, SEMA3B, LAMB2, SERPINB4 and CTF, their algorithm was able to accurately predict the outcome of IPF patients [74]. In the light of these studies, our study demonstrates the dynamicity in nanomechanical alteration of PDAC cells in the presence of collagen compared to cells cultured in the absence of collagen and the resulting signatures can be employed to accurately classify cells from the ECM in a temporal manner using ML algorithm. Herein, we empirically demonstrated that utilizing an ML-based classification algorithm, namely Support Vector Machine, can automatically classify distinct types of cells and can distinguish between cells and collagen, a type of ECM, with almost near-perfect accuracy. We observed that certain fibronectin-collagen mixtures are difficult to classify due to their almost similar AFM properties. Still, our proposed approach was able to yield 80 % accuracy in such a 1:75 mixture ratio. We believe that with more input samples, i.e., more collected data, this accuracy can be further improved. It is clear from our results that using nanomechanical properties of cells and ECM with a sophisticated ML algorithm to automatically distinguish between cells and ECM is a promising new research avenue. Such automation will open new doors for processing and analyzing so-called ‘big data’ potentially collected from thousands of patients. We acknowledge that this is a proof of principle study where only the collagen-fibronectin compositions were considered, and it is not a measure of a healthy or fibrotic microenvironment. However, simplifying the study assists us in reverse-engineering the impact of individual and mixed ECM components in PDAC paradigm. In the future, we plan to incorporate other ECM components identified in Ref. [33] in a one-at-a-time manner and truly make this a comprehensive model that will be in a true sense able to differentiate a healthy and a fibrotic microenvironment. Moreover, it has been shown previously through bulk rheology and mechanical setup experiments that ECM experiences non-linear mechanical response from the adjacent cells [75]. AFM has been proven to evaluate non-linear nanomechanical attributes of cells such as Drained Poisson's ratio, diffusion coefficient and pore size together commonly known as the photoelasticity parameters [76]. These additional parameters will add more robustness to our classification model, especially post inclusion of other cellular and ECM components in the future. And lastly, the morphological traits of ECM could potentially add an extra layer of unique signatures to enhance the ML algorithm performance. ECM has heterogeneity in topography and nanomechanical attributes. In the future, extensive nano-topography studies will also be conducted along with monitoring the nanomechanical attributes. For a robust ML algorithm, it is pivotal that various differentiating signatures are fed into the algorithm to make an informed decision. By comprehensively varying the nano-topographies of the ECM compositions, we could potentially visualize differences in their orientation, fiber length, crosslinks, etc. in the presence of various cellular entities. This could be an exciting avenue and serve as unique signatures to our ML algorithm, which will further enhance the decision-making ability of the algorithm.

This will help to better understand the complex interactions between cells and ECM. Furthermore, the knowledge of such interaction can then be leveraged for breakthroughs in therapeutic avenues targeting ECM and early cancer intervention.

## 5 Conclusion

An increase in collagen gel thickness via serial polymerization of collagen monomers was confirmed via AFM. By increasing the collagen gel thickness, its nanomechanical attributes such as stiffness and adhesion increased proportionally whereas deformation decreased correspondingly. The nanomechanical attributes of the collagen and fibronectin mixture were significantly different from collagen alone. PDAC cells and collagen mutually influenced each other's nanomechanical attributes dynamically. ML-based technique can be utilized to classify between cells and ECM components with high-end accuracy solely relying on AFM's nanomechanical attributes. Incorporating other ECM components such as elastin, laminin, and hyaluronic acid along with immune cells, pancreatic stellate cells, and fibroblasts will improve our insights regarding the PDAC desmoplasia (fibrosis) paradigm.

## Funding

This work is supported partly by 10.13039/100000002National Institutes of Health grants CA78383, CA150190, and NS129671-01, 10.13039/100000050NHLBI (#HL140411) and 10.13039/100006827Florida Department of Health (Cancer Research Chair Fund, Florida #3J) and 10.13039/100006827Florida Department of Health Grant #20K02 to DM and 10.13039/100000871Mayo Clinic Pancreatic Cancer SPORE Career Enhancement Award, Eagles fifth District Cancer Telethon−Cancer Research Fund and Jay and Deanie Stein Career Development Award for Cancer Research at 10.13039/100000871Mayo Clinic Jacksonville, 2019 Benefactor Funded Champions for Hope Pancreatic Cancer to SB.

## CRediT authorship contribution statement

**Tanmay Kulkarni:** Conceptualization, Data curation, Formal analysis, Investigation, Methodology, Writing – original draft, Writing – review & editing. **Olivia-Marie Robinson:** Data curation, Formal analysis. **Ayan Dutta:** Data curation, Formal analysis, Methodology, Software, Validation, Writing – review & editing. **Debabrata Mukhopadhyay:** Conceptualization, Funding acquisition, Methodology. **Santanu Bhattacharya:** Conceptualization, Funding acquisition, Methodology, Writing – review & editing.

## Declaration of competing interest

The authors declare that they have no known competing financial interests or personal relationships that could have appeared to influence the work reported in this paper.

## Data Availability

Data will be made available on request.
