# Supplementary material for: Machine learning-based approach for automated classification of cell and extracellular matrix using nanomechanical properties
Source: Mater Today Bio. 2024 Jan 20;25:100970. doi: 10.1016/j.mtbio.2024.100970 (PMC10835007; doi:10.1016/j.mtbio.2024.100970)
Supplement: Multimedia component 1 [file mmc1.docx]

**Supplementary information**

**Machine Learning-based Approach for Automated Classification of Cell and Extracellular Matrix Using Nanomechanical Properties**

Tanmay Kulkarni^1**^, Olivia-Marie Robinson^1^, Ayan Dutta^2^, Debabrata Mukhopadhyay^1,3^ and Santanu Bhattacharya^1,3, *^

^1^Department of Biochemistry and Molecular Biology, Mayo Clinic College of Medicine and Science, 4500 San Pablo Road South, Jacksonville, FL 32224, USA.

^2^School of Computing, University of North Florida, Jacksonville, FL, 32224 USA.

^3^Department of Physiology and Biomedical Engineering, Mayo Clinic College of Medicine and Science, 4500 San Pablo Road South, Jacksonville, FL 32224, USA.

**Corresponding authors:**

* Santanu Bhattacharya

Department of Biochemistry and Molecular Biology, Mayo Clinic College of Medicine and

Science, 4500 San Pablo Road South, Jacksonville, FL 32224, USA.

[bhattacharya.santanu@mayo.edu](mailto:bhattacharya.santanu@mayo.edu)

** Tanmay Kulkarni

Department of Biochemistry and Molecular Biology, Mayo Clinic College of Medicine and

Science, 4500 San Pablo Road South, Jacksonville, FL 32224, USA.

[Kulkarni.tanmay@mayo.edu](mailto:Kulkarni.tanmay@mayo.edu)

**Supplementary figures**


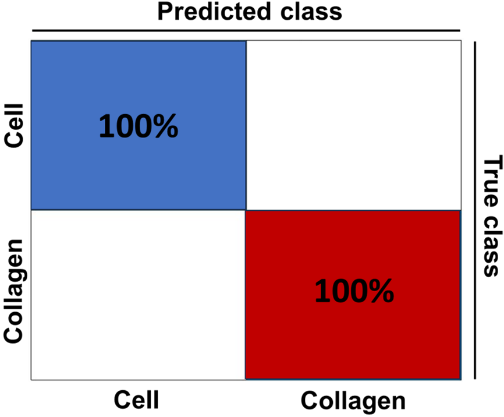


**Figure S1. ML technique classifies cellular and collagen regions based on nanomechanical attributes using an SVM model with a linear kernel.**


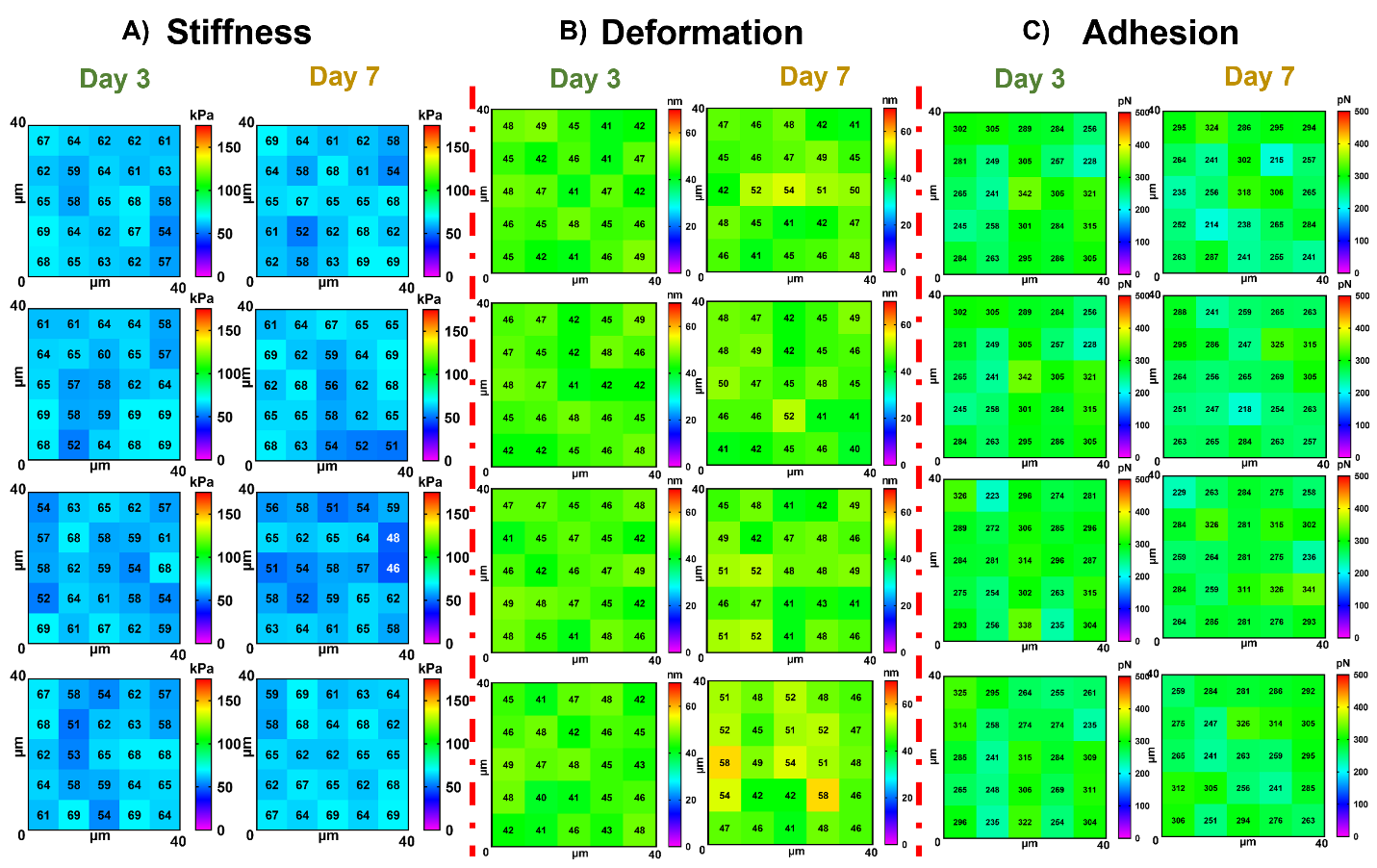


**Figure S2. Heat map displaying dynamic alteration of nanomechanical collagen attributes without PDAC cells.** A) Stiffness. B) Deformation. C) Adhesion.


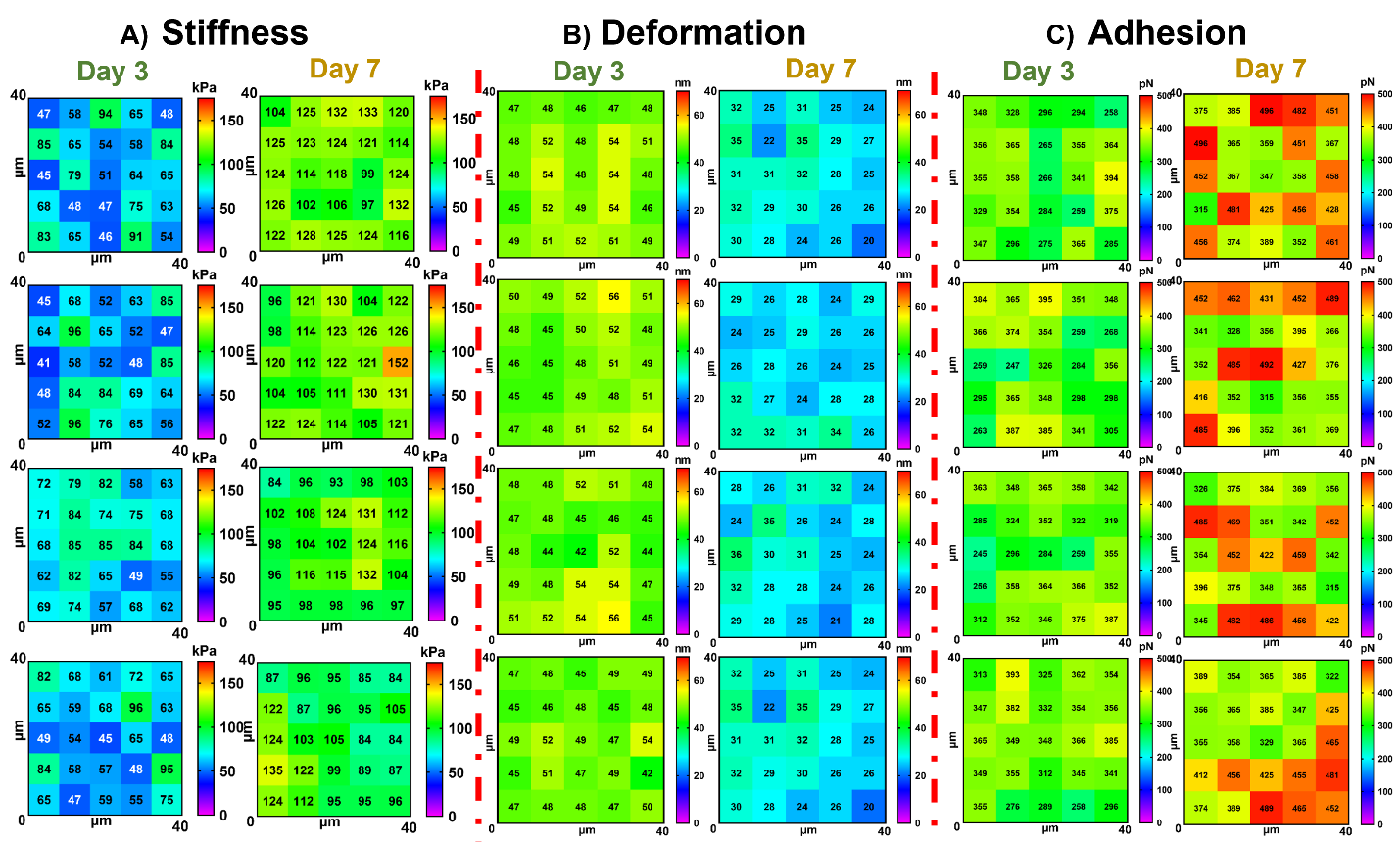


**Figure S3. The heat map shows dynamic alteration of nanomechanical collagen attributes in Panc-1 cells' presence.** A) Stiffness. B) Deformation. C) Adhesion.


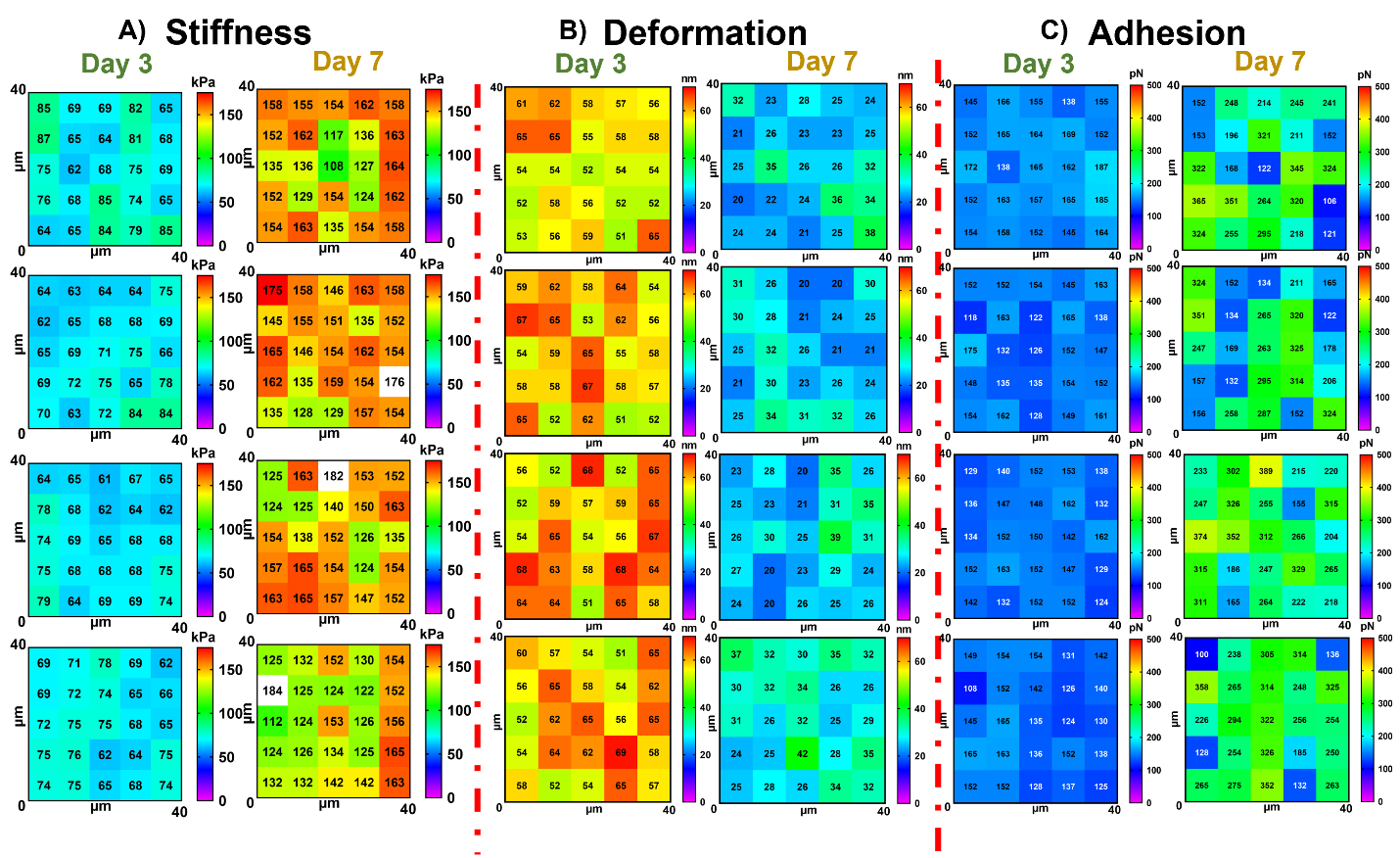
**Figure S4. Heat map displaying dynamic alteration of nanomechanical attributes of collagen in the presence of AsPC-1 cells. A) Stiffness. B) Deformation. C) Adhesion.**
